# Percutaneous coronary intervention with second-generation drug-eluting stent versus bare-metal stent: Systematic review and cost–benefit analysis

**DOI:** 10.1371/journal.pone.0177476

**Published:** 2017-05-12

**Authors:** Thomas G. Poder, Jihane Erraji, Lucien P. Coulibaly, Kouamé Koffi

**Affiliations:** 1 UETMIS and CRCHUS, CIUSSS de l’Estrie-CHUS, Sherbrooke, Québec, Canada; 2 Département d’Économique and GREDI, École de Gestion, Université de Sherbrooke, Sherbrooke, Québec, Canada; 3 CERDI, Université d’Auvergne, Clermont-Ferrand, France; 4 École de réadaptation, FMSS, Université de Sherbrooke, Sherbrooke, Québec, Canada; 5 Département de santé publique, UFR Sciences Pharmaceutiques, Université Félix Houphouët Boigny, Abidjan, Côte d’Ivoire; University of Tampere, FINLAND

## Abstract

**Background:**

Drug-eluting stents (DESs) were considered as ground-breaking technology promising to eradicate restenosis and the necessity to perform multiple revascularization procedures subsequent to percutaneous coronary intervention. Soon after DESs were released on the market, however, there were reports of a potential increase in mortality and of early or late thrombosis. In addition, DESs are far more expensive than bare-metal stents (BMSs), which has led to their limited use in many countries. The technology has improved over the last few years with the second generation of DESs (DES-2). Moreover, costs have come down and an improved safety profile with decreased thrombosis has been reported.

**Objective:**

Perform a cost–benefit analysis of DES-2s versus BMSs in the context of a publicly funded university hospital in Quebec, Canada.

**Methods:**

A systematic review of meta-analyses was conducted between 2012 and 2016 to extract data on clinical effectiveness. The clinical outcome of interest for the cost–benefit analysis was target-vessel revascularization (TVR). Cost units are those used in the Quebec health-care system. The cost–benefit analysis was based on a 2-year perspective. Deterministic and stochastic models (discrete-event simulation) were used, and various risk factors of reintervention were considered.

**Results:**

DES-2s are much more effective than BMSs with respect to TVR rate ratio (i.e., 0.29 to 0.62 in more recent meta-analyses). DES-2s seem to cause fewer deaths and in-stent thrombosis than BMSs, but results are rarely significant, with the exception of the cobalt–chromium everolimus DES. The rate ratio of myocardial infraction is systematically in favor of DES-2s and very often significant. Despite the higher cost of DES-2s, fewer reinterventions can lead to huge savings (i.e., -$479 to -$769 per patient). Moreover, the higher a patient’s risk of reintervention, the higher the savings associated with the use of DES-2s.

**Conclusion:**

Despite the higher purchase cost of DES-2s compared to BMSs, generalizing their use, in particular for patients at high risk of reintervention, should enable significant savings.

## Introduction

There have been quite a few major revolutions in interventional cardiology in recent decades. The first revolution occurred with the introduction of percutaneous balloon angioplasty; the second with that of the bare-metal stent (BMS). Unlike the balloon, which widens a narrowed segment in the target vessel, the BMS is a hollow cylinder made of fine metal mesh (stainless steel or an alloy) inserted into a coronary artery to maintain the patency of weakened arterial segments after balloon angioplasty [1]. These interventions have made major clinical improvements possible and have very significantly reduced reintervention rates [2]. However, the restenosis rate with BMS remains high due to in-stent restenosis related to neointimal hyperplasia. A new type of stent was developed to attenuate this situation: the drug-eluting stent (DES). This stent type is designed to gradually release an antiproliferative drug in the vessel being treated [3]. The medication is contained in a polymer coating on the stent's metal surface (sirolimus or paclitaxel for first-generation stents and everolimus or zotarolimus for second-generation ones). Drug-eluting stents (DESs) have undergone many clinical trials in order to determine their efficacy and safety with respect to BMSs for percutaneous coronary intervention (PCI). While the findings suggest that DESs are superior to BMSs with respect to the target lesion or vessel revascularization rate, the results are conflicting in terms of the risk of thrombosis and the mortality rate [2]. Safety does appear to have been improved with the second generation of DESs (DES-2) [4]. Moreover, until recently, the cost of purchasing DESs was relatively much higher than that of BMSs, which limited DES generalization. Given the recent changes in the data on the efficacy, safety, and cost of DESs with respect to BMSs, there is a need to establish the cost–benefit ratio to assess whether their use should be generalized to eligible patients.

## Objective

Perform a cost–benefit analysis of DES-2s versus BMSs in the context of a publicly funded university hospital in Quebec, Canada.

## Methods

### Systematic review

The methodology adopted was a systematic review of systematic reviews with meta-analysis (S1 Table). A review protocol was developed in which Pubmed and the Web sites of the Cochrane Library, the CADTH and INESSS assessment agencies, the Center for Reviews and Dissemination (CRD), and health technology assessment (HTA) units in university hospital centers in Quebec (i.e., McGill, CHUM, CHUQ-UL, and Ste-Justine) were consulted. The literature review was completed by reading the references in the articles included. The reference period was from January 1, 2012 to August 17, 2016. This reference period was selected in order to get the most recent evidence and to avoid the repetition of older studies already included in the most recent meta-analyses.

The keywords used singly or in combination were: drug-eluting stent, coronary-artery disease, percutaneous coronary intervention, coronary restenosis, target-vessel revascularization. In the case of PubMed, these keywords yielded the following search query: ("Drug-Eluting Stents" [Mesh]) AND ("Coronary Artery Disease" [Mesh] OR "Percutaneous Coronary Intervention" [Mesh] OR "Coronary restenosis" [Mesh] OR "target vessel revascularization"). In PubMed, we also selected the "Humans" and "Systematic reviews" filters.

In order to be included, the studies identified had to be systematic reviews with meta-analysis, deal with percutaneous coronary interventions for patients with a coronary artery disease, and compare second-generation DESs with BMSs. Studies combining the results for multiple generations of DESs were excluded, as were studies combining second-generation DESs with bioabsorbable DESs and those dealing with treatment of in-stent restenosis. No language criteria were used.

Groups of two or three researchers read the titles and abstracts identified in the databases. Once this preliminary step had been completed, pairs of researchers read the full articles and selected articles based on the inclusion and exclusion criteria. A third researcher served as arbitrator if disagreements arose. Two researchers extracted the data and entered them into an Excel spreadsheet. At this step, the collected data were compared. In cases of divergences, a third researcher would read the study in question and decide the issue. The quality of each study was assessed with the AMSTAR checklist [5]. The main variables collected were target-vessel revascularization (TVR) rate, mortality rate, in-stent thrombosis rate, and myocardial-infarction rate. The measure was the rate ratio.

### Cost–benefit analysis

The clinical data yielded by the systematic literature review were combined with our institution's cost data. The study perspective adopted is that of Quebec's public-health-care system and costs are expressed in 2016 Canadian dollars. The cost difference between the DES-2s and BMS based on their relative clinical effectiveness was calculated on the basis of 1000 patients receiving PCI. Target-vessel revascularization rate was selected as the efficacy variable because it is one of the most documented. In addition, it has the advantage of producing unequivocal results when DES-2s are compared with BMSs. Moreover, this variable can be used to calculate reintervention costs avoided for failed vascularization and therefore for conducting a cost–benefit analysis. The time horizon was two years. This time frame was selected because it has been relatively well documented with respect to longer periods. Moreover, it made it possible to cover most of the occurrences of failed revascularizations (i.e., more than half of all reinterventions occur within the first two years). A sensitivity analysis was conducted, taking into consideration an interval of several values related to the relative efficacy of DESs compared to BMSs. Similarly, several simulations have been carried out taking in consideration different groups of patients that would benefit from DES-2s. These groups of patients were determined based on risk factors of failed vascularization subsequent to stent implantation. The risk factors cited most frequently in the literature are vessel diameter (<3 mm), lesion length (≥20mm), patient's diabetic status, and a history of PCI or coronary-artery bypass grafting [6,7]. The cost–benefit simulations were based on deterministic and stochastic models. The deterministic models were run based on Excel (Microsoft Corp., USA). Discrete-event simulation with Arena^®^ (Rockwell software, USA) was used for the stochastic models. This method was selected because it can generate a random distribution of patients while considering the possibility that they have different characteristics (i.e., risk factors). It can also introduce different values for rate ratios for each of the years considered. A total of 1000 replications was performed for each simulation with the discrete-event simulation method. Given the short time frame assessed, no discount rate was used herein.

## Results

**Fig 1:**
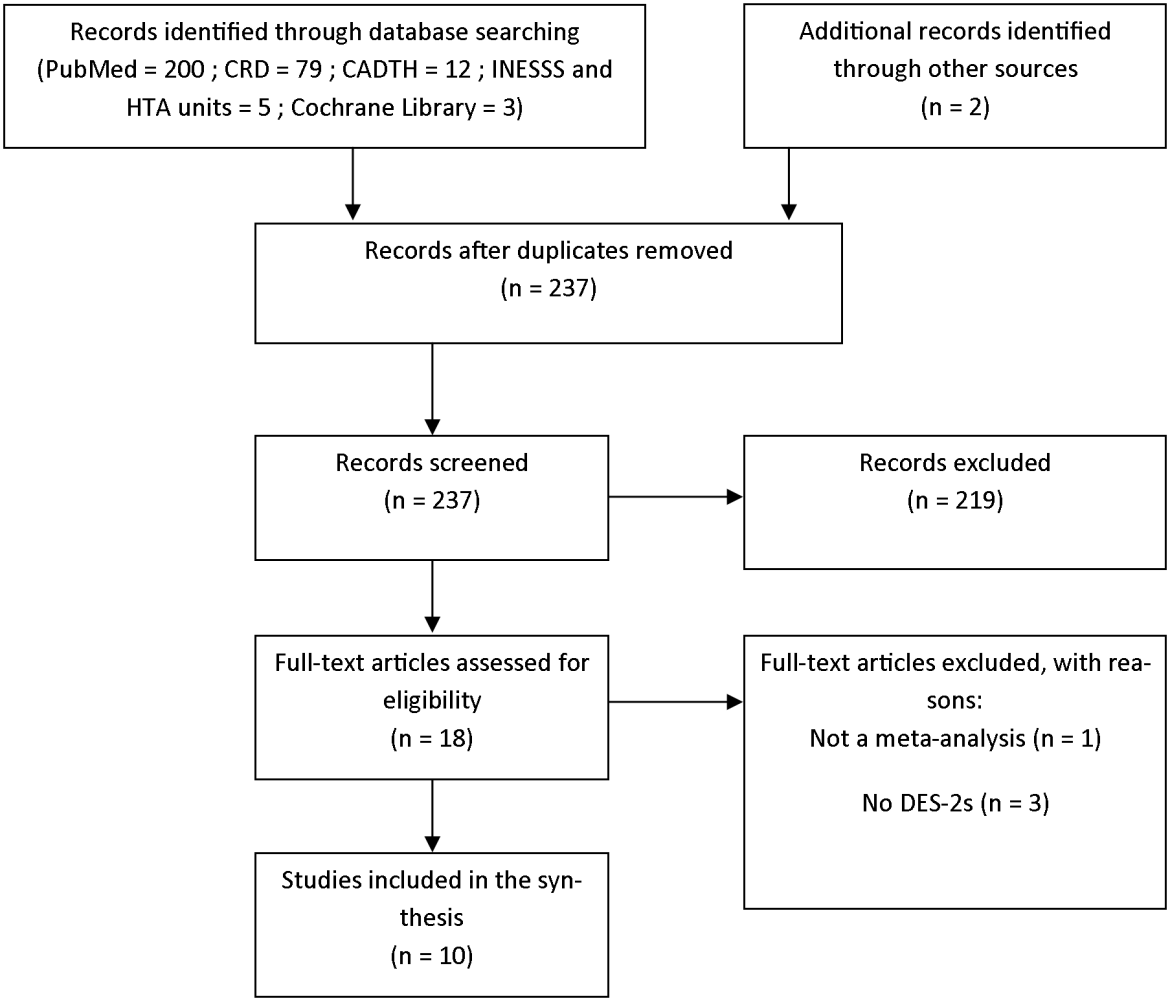
PRISMA flow diagram (17 august 2016).

Our searching produced 237 studies without duplications. Of these, 18 were selected to be read in full and 10 were included. The findings of our literature-search strategy are presented in a PRISMA flow chart (Fig 1).

Of the 10 studies included, 2 were conventional meta-analyses, 7 network meta-analyses, and 1 meta-analysis with patient-level data (Table 1). The quality of the studies was deemed very good with AMSTAR scores between 9 and 10.5. The authors of these 10 studies all declared their conflicts of interest and only 3 did not perform an assessment of publication bias.

**Table 1:** Characteristics of meta-analyses included.

Authors	Kind of MA	Period	Stent and generation	Nb. Trials included	Nb. patients	Characteristics of patients	AMSTAR score
[8]	Network MA	Up to April 2012	BMS, DES-1, DES-2	42 RCT	10,714 patients	Diabetic patients with coronary-artery disease and primary PCI	10/11
[9]	Network MA	Up to March 2012	BMS, DES-1, DES-2	76 RCT	57,138 patients	Patients with coronary-artery disease and primary PCI	9.5/11
[10]	Network MA	Up to March 2013	BMS, DES-1, DES-2	28 RCT	14,740 patients	Patients with STEMI	9.5/11
[6]	MA	Up to March 2012	BMS, DES-1, DES-2	6 RCT	4,393 patients	Patients with coronary-artery disease and large vessels (> = 3 mm)	10/11
[4]	Network MA	2002 to 2011	BMS, DES-1, DES-2	49 RCT	50,844 patients	Patients with coronary-artery disease	10/11
[11]	Network MA	Up to 2012	BMS, DES-1, DES-2	22 RCT	12,453 patients	Patients with STEMI and primary PCI	10/11
[12]	Network MA	Up to 2013	BMS, DES-1, DES-2, BS	89 RCT	85,490 patients	Patients with coronary-artery disease	10.5/11
[13]	Network MA	Up to 2014	BMS, DES-1, DES-2, BS	51 RCT	52,158 patients	Patients with coronary-artery disease	10.5/11
[14]	MA with patient-level data	Up to December 2013	BMS, EES	5 RCT	4,896 patients	Patients with coronary-artery disease	9/11
[15]	MA	Up to July 2013	BMS, DES-1, DES-2	10 RCT	7,592 patients	Patients with STEMI	10/11

Notes: MA: Meta-analysis, BMS: Bare-metal stent, DES: Drug-eluting stent (generation 1 or 2), BS: Bioabsorbable stent, EES: Everolimus-eluting stent, RCT: randomized controlled trial, PCI: Percutaneous coronary intervention, STEMI: ST-segment elevation myocardial infraction.

### Stents used

These meta-analyses compared second-generation DESs to BMSs, either directly or indirectly (i.e., network meta-analyses). The DESs inventoried are the cobalt-chromium everolimus-eluting stent (Co-Cr-EES), platinum-chromium everolimus-eluting stent (Pt-Cr-EES), Resolute Integrity zotarolimus-eluting stent (Re-ZES), and the polymer-coated zotarolimus-eluting stent (PC-ZES or phophorylcholine-polymer-ZES). Some studies did not specify the DES type; only the eluant (i.e., everolimus or zotarolimus).

### Efficacy

#### Target-vessel revascularization

With the exception of zotarolimus stents—the PC-ZES in particular, which is less effective—the meta-analyses overall revealed a significant reduction in TVR in patients who received a DES-2 instead of a BMS (Table 2). The TVR ratio rates were between 0.3 and 0.45 in the most recent studies on EESs and the Re-ZES, with values frequently around 0.4. The ratio rates for the PC-ZES were between 0.5 and 0.62. Moreover, the reduction in TVR rate was higher with the platinum-chromium alloy than with the chromium-cobalt alloy [12,13].

**Table 2:** Rate ratio of target-vessel revascularization (TVR).

Authors	Comparator	Target-vessel revascularization		
		1 year	2 years	≥ 3 years
[8] *	EES vs. BMS		**0.31 [0.19–0.47]**	
	ZES vs. BMS		**0.63 [0.42–0.96]**	
[9]	EES vs. BMS	**0.28 [0.21–0.37]**	**0.39 [0.31–0.48]**	
	ZES vs. BMS	**0.48 [0.36–0.66]**	**0.61 [0.48–0.77]**	
	Re-ZES vs. BMS	**0.31 [0.17–0.57]**	**0.44 [0.27–0.68]**	
[10] *	EES vs. BMS		**0.42 [0.26–0.62]**	
	ZES vs. BMS		0.96 [0.43–1.87]	
	Re-ZES vs. BMS		0.26 [0.04–1.71]	
[6]	EES vs. BMS	**0.34 [0.22–0.52]**		
[11]	EES-Co-Cr vs. BMS	**0.45 [0.29–0.66]**	**0.43 [0.28–0.62]**	
	PC-ZES vs. BMS	0.60 [0.34–1.05]	0.67 [0.40–1.16]	
[12]	EES-Pt-Cr vs. BMS	**0.30 [0.15–0.59]**	**0.34 [0.20–0.60]**	
	EES-Co-Cr vs. BMS	**0.30 [0.23–0.39]**	**0.39 [0.32–0.48]**	
	Re-ZES vs. BMS	**0.33 [0.19–0.58]**	**0.42 [0.27–0.66]**	
	PC-ZES vs. BMS	**0.56 [0.40–0.77]**	**0.62 [0.49–0.80]**	
[13]	EES-Pt-Cr vs. BMS			**0.34 [0.19–0.57]**
	EES-Co-Cr vs. BMS			**0.40 [0.32–0.49]**
	Re-ZES vs. BMS			**0.45 [0.29–0.68]**
	PC-ZES vs. BMS			**0.50 [0.41–0.62]**
[14]	EES vs. BMS		**0.29 [0.20–0.41]**	
[15] **	EES vs. BMS	**0.55 [0.35–0.86]**		

Notes: BMS: Bare-metal stent, EES: Everolimus-eluting stent, ZES: Zotarolimus-eluting stent,

* Results at 6 months or more (up to 5 years)

** Results at 1 year or more; significant at 5% in bold.

### Safety

#### Mortality

No statistically significant difference was observed with respect to total mortality (i.e., all causes) in the 7 meta-analyses reporting on this issue (Table 3). Most of the DES-2s nevertheless were associated with a downward trend in mortality, particularly with Co-Cr-EESs. Moreover, while Palmerini et al. [12] observed no decreased mortality related to cardiac issues, Valgimigli et al. [14] indicated a lower mortality rate with Co-Cr-EES than with BMS (0.69; CI 0.50–0.94).

**Table 3:** Rate ratio of mortality (all causes).

Authors	Comparator	Mortality ≤ 1 year	Mortality 2 years	Mortality 3 years
[8] *	EES vs. BMS		0.83 [0.42–1.46]	
	ZES vs. BMS		1.14 [0.58–2.27]	
[9]	EES vs. BMS	0.87 [0.64–1.16]	0.81 [0.64–1.01]	
	ZES vs. BMS	1.28 [0.85–1.80]	0.94 [0.73–1.24]	
	Re-ZES vs. BMS	0.66 [0.36–1.18]	0.71 [0.31–1.09]	
[10] *	EES vs. BMS		0.78 [0.48–1.24]	
	ZES vs. BMS		1.52 [0.70–2.96]	
[11]	EES-Co-Cr vs. BMS	0.72 [0.48–1.02]	0.69 [0.48–1.02]**	
	PC-ZES vs. BMS	1.22 [0.73–2.09]	1.29 [0.76–2.15]**	
[12]	EES-Pt-Cr vs. BMS	0.88 [0.48–1.67]	0.86 [0.54–1.37]**	
	EES-Co-Cr vs. BMS	0.82 [0.63–1.06]	0.83 [0.68–1.01]**	
	Re-ZES vs. BMS	0.60 [0.37–1.01]	0.68 [0.45–1.01]**	
	PC-ZES vs. BMS	1.19 [0.78–1.79]	0.95 [0.76–1.16]**	
[13]	EES-Pt-Cr vs. BMS			0.73 [0.40–1.40]
	EES-Co-Cr vs. BMS			**0.81 [0.64–1.00]**
	Re-ZES vs. BMS			0.79 [0.52–1.20]
	PC-ZES vs. BMS			0.88 [0.70–1.10]
[14]	EES-Co-Cr vs. BMS		0.84 [0.66–1.07]	

Notes: BMS: Bare-metal stent, EES: Everolimus-eluting stent, ZES: Zotarolimus-eluting stent,

* Results at 6 months or more (up to 5 years)

** Results at 1 year or more; significant at 5% in bold.

#### Myocardial infarction

Myocardial infarction is an immediate consequence of occlusion of a coronary artery or one of its branches. Nearly all of the studies with infarction occurring at one year or less indicated a significant drop in the myocardial-infarction rate (Table 4). The lone exception was PC-ZES in the Palmerini et al. study [11]. Nevertheless, more recent meta-analyses conducted by Palmerini et al. [12,13] contain new data indicating that the PC-ZES reduces the risk of myocardial infarction at less than one year and also at more than one year. The meta-analysis results at more than one year were more mixed. While a downward trend in myocardial-infarction rate was indicated with all the DES-2s, only the results associated with Co-Cr-EESs and PC-ZESs were statistically significant [13].

**Table 4:** Rate ratio of myocardial infarction.

Authors	Comparator	Myocardial infarction ≤ 1 year	Myocardial infarction > 1 year
[8] *	EES vs. BMS		0.52 [0.21–1.09]
	ZES vs. BMS		2.16 [0.91–8.45]
[9]	EES vs. BMS	**0.55 [0.41–0.73]**	**0.63 [0.51–0.80]**
	ZES vs. BMS	**0.66 [0.49–0.84]**	**0.69 [0.52–0.89]**
	Re-ZES vs. BMS	**0.55 [0.38–0.82]**	0.69 [0.45–1.03]
[10] *	EES vs. BMS		0.63 [0.28–1.39]
	ZES vs. BMS		0.39 [0.10–1.43]
	Re-ZES vs. BMS		0.38 [0.04–2.97]
[11]	EES-Co-Cr vs. BMS	**0.55 [0.34–0.93]**	0.66 [0.44–1.05]
	PC-ZES vs. BMS	0.58 [0.31–1.03]	0.68 [0.36–1.24]
[12]	EES-Pt-Cr vs. BMS	**0.42 [0.23–0.78]**	**0.56 [0.30–1.00]**
	EES-Co-Cr vs. BMS	**0.57 [0.45–0.72]**	**0.66 [0.52–0.78]**
	Re-ZES vs. BMS	**0.56 [0.37–0.85]**	**0.67 [0.46–0.96]**
	PC-ZES vs. BMS	**0.65 [0.48–0.89]**	**0.71 [0.57–0.87]**
[13] **	EES-Pt-Cr vs. BMS		0.62 [0.29–1.20]
	EES-Co-Cr vs. BMS		**0.66 [0.52–0.85]**
	Re-ZES vs. BMS		0.65 [0.42–1.02]
	PC-ZES vs. BMS		**0.77 [0.60–0.96]**
[14]	EES-Co-Cr vs. BMS		**0.71 [0.55–0.93]**

Notes: BMS: Bare-metal stent, EES: Everolimus-eluting stent, ZES: Zotarolimus-eluting stent,

* Results at 6 months or more (up to 5 years)

** Results at 3.8 years; significant at 5% in bold.

#### Stent thrombosis

Although rare (<2%–3%), stent thrombosis is a devastating complication and associated with high rates of mortality and morbidity [4]. The definition of thrombosis includes angiographically demonstrated thromboses that can occur within hours of implantation (acute), during the first month (subacute), during the first year (late), and beyond (very late). The results reported in Table 5 provide the rates of definite or probable thromboses according to Academic Research Consortium criteria [16]. Except in the case of PC-ZESs, the reduction in the rate of subacute thromboses (≤30 days) was statistically significant, regardless of the type of DES-2. Only the Co-Cr-EESs evidenced a reduction in the rate of late (>30 days) and/or very late (>1 year) thromboses. The other DES-2s only indicated a downward trend in this rate compared to BMSs.

**Table 5:** Rate ratio of definite or probable thrombosis.

Authors	Comparator	Thrombosis ≤ 30d	Thrombosis > 30d	Thrombosis 1 year	Long-term total thrombosis (> 1 year)
[8]	EES vs. BMS		0.43 [0.13–1.23]		0.96 [0.03–24.33]
	ZES vs. BMS		4.08 [0.82–20.58]		4.09 [0.15–143.90]
[9]	EES vs. BMS			**0.41 [0.22–0.72]**	**0.46 [0.31–0.70]**
	ZES vs. BMS			1.04 [0.53–1.98]	0.69 [0.39–1.28]
	Re-ZES vs. BMS			0.61 [0.19–1.72]	0.62 [0.29–1.44]
[10]	EES vs. BMS			0.89 [0.09–8.67]	**0.39 [0.18–0.82]**
	ZES vs. BMS			133.10 [0.56–12540]	0.69 [0.23–1.60]
[4]	EES-Pt-Cr vs. BMS	**0.08 [0.00–0.96]**	NA	0.34 [0.05–2.12]	NA
	EES-Co-Cr vs. BMS	**0.32 [0.17–0.60]**	**0.42 [0.17–0.95]**	**0.34 [0.21–0.53]**	0.92 [0.25–4.95]
	Re-ZES vs. BMS	**0.32 [0.09–0.99]**	1.70 [0.33–10.83]	0.53 [0.21–1.26]	0.96 [0.07–18.52]
	PC-ZES vs. BMS	1.17 [0.53–2.72]	2.10 [0.60–9.20]	1.13 [0.60–2.11]	0.91 [0.13–9.25]
[11]	EES-Co-Cr vs. BMS	**0.28 [0.12–0.61]**	0.73 [0.11–6.46]	**0.36 [0.18–0.66]**	**0.41 [0.16–0.88]**
	PC-ZES vs. BMS	0.45 [0.15–1.12]	0.26 [0.00–4.95]	0.47 [0.19–1.04]	0.68 [0.21–1.80]
[12]	EES-Pt-Cr vs. BMS	**0.16 [0.03–0.64]**	3.23 [0.26->99]	0.35 [0.12–1.03]	0.43 [0.16–1.03]
	EES-Co-Cr vs. BMS	**0.32 [0.18–0.57]**	**0.44 [0.20–0.92]**	**0.39 [0.27–0.57]**	**0.48 [0.34–0.70]**
	Re-ZES vs. BMS	**0.32 [0.10–0.90]**	2.28 [0.47–12.88]	0.59 [0.28–1.28]	0.73 [0.35–1.55]
	PC-ZES vs. BMS	0.73 [0.38–1.43]	2.02 [0.76–5.89]	0.99 [0.62–1.55]	0.75 [0.50–1.12]
[13]	EES-Pt-Cr vs. BMS				0.58 [0.11–2.70]*
	EES-Co-Cr vs. BMS				0.50 [0.33–0.73]*
	Re-ZES vs. BMS				0.81 [0.34–1.70]*
	PC-ZES vs. BMS				0.66 [0.44–1.10]*
[14]	EES-Co-Cr vs. BMS				**0.48 [0.31–0.73]**
[15]	EES vs. BMS				**0.37 [0.15–0.87]**

Notes: BMS: Bare-metal stent, EES: Everolimus-eluting stent, ZES: Zotarolimus-eluting stent, NA: Not available

* Results at 3.8 years; significant at 5% in bold.

### Cost–benefit analysis

The cost to the Quebec health-care system for reintervention subsequent to failed vascularization was $11,167.83 as of July 1, 2016 (Table 6). This cost was calculated based on the supplies required to perform a PCI (82%) or coronary-artery bypass grafting (18%), remuneration of health-care personnel, cost of the dual antiplatelet therapy delivered by the pharmacy, cost of an intermediate-care bed, cost of cardiac rehabilitation, laboratory analyses, and overhead related to operating health-care institutions (hereafter referred to as support services).

**Table 6:** Cost of reintervention following a revascularization failure in 2016.

	Cost for the Quebec health-care system
Health-care professionals	319.20
Medical doctors	792.43
Supplies	2256.02
Dual antiplatelet therapy	670.43
Intermediate care	4077.99
Cardiac rehabilitation	1575.00
Laboratory analysis	25.49
Support services	1451.27
**Total**	**$11,167.83**

Notes: The reintervention consists of PCI in 82% and coronary-artery bypass grafting (CABG) in 18%. Costs are in 2016 Canadian dollars.

The additional cost associated with implanting a DES-2 compared to a BMS was calculated based on the purchase price of these two stents by our institution and their rate of use in the hemodynamics laboratory. Prior to 2013, the additional cost to our institution for DES-2s instead of BMSs was nearly $1000. In 2013, our suppliers significantly decreased their prices, reducing the cost differential to $210. In 2016, a subsequent decrease in price was negotiated, reducing the cost differential to $190. Given that each PCI requires installing an average of 1.6 stents [14], the additional cost per intervention is $304.

We used two TVR rate ratios for our cost–benefit simulations. To begin, we used a rate ratio of 0.4 to 2 years estimated in the meta-analysis by Palmerini et al. [12]. This value falls within the interval of values inventoried in our systematic review and is also very close to the rate ratio estimated by Bangalore et al. [10] for EESs. We also considered the rate ratio of 0.3 provided in the only meta-analysis using individual patient data, that is, that of Valgimigli et al. [14]. These two values enabled us to carry out a sensitivity analysis. In our discrete-event simulations, we set two different rate ratios for each of the two years of follow-up. The simulations considering the rate ratio provided by Palmerini et al. [12] yielded a value of 0.35 at 1 year and another of 0.45 at the second year. For the simulations using the value in Valgimigli et al. [14], we left the rate ratio at 0.3 since no lower values have been observed so far in other meta-analyses.

In building our patient sample, we decided to use the populational data in the Tu et al. study [17]. Theirs was a cohort study involving a general population in Ontario, Canada, with coronary-artery disease. Since Ontario neighbors on Quebec, we deemed that it would be more representative of the situation of the patients at our institution than the populations used in the randomized trials, which are subject to very specific criteria selection. Moreover, it is one of the rare studies that provide the proportion of patients at risk of TVR at 2 years and quantify their risk ratio. The proportions of patients with different combinations of vessel diameter and lesion length therefore came from the Tu et al. study [17]. As for the proportion of diabetics, we used the value provided by our institution's InfoCentre, namely 25%. With respect to the proportion of patients who had already had a PCI or bypass surgery, we adopted a rate of 15% based on the statistics provided by our institution's InfoCentre as well as on information found in the studies conducted by Tu et al. [17] and Taniwaki et al. [7]. In total, that enabled us to construct 16 groups of patients with different proportions and different risk levels. For each of the groups, a TVR rate at two years was assigned depending on whether the patient was in a DES-2 or BMS group. The TVR rate for the BMS groups were provided by the Tu et al. study [17]. Since the Tu et al. study [17] didn't consider the risk factor for a previous PCI or coronary-artery bypass grafting, we applied a 2.02 correction factor based on the findings in the study conducted by Taniwaki et al. [7,18]. The risk factor associated with diabetes was 1.37; 1.69 for a small vessel; 1.35 for a long vessel [17].

Table 7 provides the number of reinterventions avoided per 1000 patients undergoing PCI. The last column of this table provides the net cost per 1000 patients (additional cost or savings). The cost difference between a patient receiving a PCI with DES-2 or with BMS can be determined by simply dividing the figure by 1000.

**Table 7:** Cost–benefit simulations over 2 years for 1000 patients.

Simulation	TVR rate		Reinterventions avoided after 1^st^ PCI	Reinterventions avoided after 1^st^ and 2^nd^ PCI	Net cost for 1000 patients ($)
	BMS	DES-2			
All patients eligible for a DES-2 (100%)					
Deterministic simulations					
RR 0.4	11.20%	4.48%	67.19	71.71	-483,204
RR 0.3	11.20%	3.36%	78.39	84.54	-629,884
Stochastic simulations					
RR 0.4	11.38%	4.66%	67.23	71.75	-479,163
RR 0.3	11.44%	4.14%	72.97	78.30	-552,422
Patients eligible for a DES-2 with at least 1 risk factor (87%)					
Deterministic simulations					
RR 0.4	12.01%	4.80%	72.05	77.25	-544,080
RR 0.3	12.01%	3.60%	84.06	91.13	-702,780
Stochastic simulations					
RR 0.4	12.17%	5.02%	71.52	76.64	-533,527
RR 0.3	12.22%	4.47%	77.50	83.51	-610,344
Patients eligible for a DES-2 with at least 2 risk factors^1^ (52%)					
Deterministic simulations					
RR 0.4	14.95%	5.98%	89.68	97.72	-769,192
RR 0.3	14.95%	4.48%	104.63	115.58	-973,113
Stochastic simulations					
RR 0.4	15.17%	6.70%	84.69	91.86	-702,415
RR 0.3	15.26%	5.97%	92.91	101.55	-810,748

Note: A negative value represents a savings.

^1^ With a minimum of two risk factors or with a previous PCI or coronary-artery bypass grafting.

Table 7 also presents the simulation results for different patient groups. The first group includes all the patients eligible for a DES-2. The second group excludes eligible patients not presenting any of the four risk factors cited above. The third group excludes all eligible patients who had not had a PCI or coronary-artery bypass grafting or who had fewer than two risk factors.

The results indicate a minimal savings of $479.16 per patient if all the patients eligible received a DES-2 and savings of up to $973.11 per patient if the DES-2s were reserved solely for the patients at greatest risk. As a percentage of required costs for a PCI, that represents savings ranging from 4.29% to 8.71%. It would thus appear that using DES-2s rather than BMSs would be much more economical given the actual price difference.

## Discussion

The studies included were of good quality as indicated by the AMSTAR scores. Nevertheless, the results obtained might be subject to some methodological bias and publication bias, which could limit confidence in the results.

First of all, the number of patients included in the meta-analyses varied greatly (from 4,896 to 85,490). Moreover, not all of the patients in them were included in the comparison of the DES-2s and BMSs. The meta-analysis in Geng et al. [6] is an extreme case in point in which one study out of the six included was used to provide data to compare EESs to BMSs. The statistical power required to confirm the observed results is therefore not adequate, particularly in the oldest meta-analyses, as indicated by Palmerini et al. [4]. The meta-analysis inclusion criteria also reveal that the same randomized controlled trials were often used from one meta-analysis to the next. That is why we deem it relevant to grant greater importance to the most recent meta-analysis. Moreover, the most recent meta-analyses include new data, making it possible to observe the evolution of DES-2 efficacy and safety over time. Indeed, they bring out that the DES-2s are even more efficacious than the BMSs than would have been suspected based on the oldest meta-analyses and that DES-2 safety data are increasingly convincing.

Despite the use of randomized trial specifications, sampling bias might have occurred in the meta-analyses that included small primary studies. Moreover, given their size, small studies are more likely to select variables that better highlight their results [19]. In addition, we noted an imbalance between the studies in certain meta-analyses, in which the size of some would have had considerable influence on the final results [6].

One of the general limitations of the meta-analyses was not adjusting the results according to patient characteristics. In this regard, Bangalore et al. [9] reported the possible existence of confounding variables that could skew the results. Adjusting the results require clinical and sociodemographic data not always yielded by the randomized clinical trials. One way to resolve this issue is to proceed like Valgimigli et al. [14] and conduct a meta-analysis based on the individual data from the randomized clinical trials. Moreover, that can bolster the statistical power of the results.

Duration of dual antiplatelet therapy is one of the main confounding variables that could affect the results, particularly the thrombosis rates. In this regard, Valgimigli et al. [14], who used treatment duration of less than or greater than a year as a control variable, indicated that treatment duration had no effect on thrombosis rate after a year. Other confounding variables were also tested in this meta-analysis; taking them into account had no impact on the results. Consequently, this bias appears to be limited.

The type of follow-up could result in an overestimation of DES-2 efficacy compared to BMSs. The follow-up in clinical trials is most often angiography, whereas the everyday reality is frequently clinical follow-up. The randomized trials therefore might have overestimated the number of reinterventions avoided with the use of DESs [6]. Moreover, patient selection in the randomized trials was relatively strict, which could have favored selection of patients who were potentially more likely to react favorably to DESs than to BMSs. Overly strict patient selection also constitutes an impediment to generalization of the results in terms of their external validation. The lack of a double-blind approach might have introduced an unfavorable bias with respect to BMSs in that the physicians might have been more aware of the restenosis risk associated with BMSs and favored more reinterventions in this group [20]. Moreover, Kalesan et al. [21] tested the sensitivity of results in terms of the method used in randomized trials, bringing to light some disparities.

It should be noted that most of the meta-analyses were funded publicly, unlike the randomized trials that the meta-analyses took up. This leads to a risk of overestimating DES effects, especially in the selection of results presented for publication. In addition, there is the usual risk of publication bias. In this respect, the meta-analysis conducted by Kalesan et al. [21] indicated a potential publication bias for small studies (fewer than 300 patients) with positive TVR results.

A final limitation of the meta-analyses inventoried is that most were network meta-analyses. Considering the small number of studies in which DES-2s and BMSs were directly compared, it appears that their results came very largely from indirect comparisons [8–10,13]. With indirect comparisons, there is the potential for bias insofar as the groups compared do not have the same characteristics at the outset. That notwithstanding, it is impossible to determine if that enhances or diminishes the estimated efficacy of DES-2s with respect to BMSs.

For their part, our cost estimations are, of course, limited by the fact that we calculated an average cost per patient and not an individual cost for each patient. Likewise, they are based on the costs in our institution, which limits generalization. That notwithstanding, we were able to consider different patient groups based on the four main risk factors for reintervention, which introduced some variability. Moreover, we conducted discrete-event simulations with 1000 replications, which made it possible to take into consideration the effect of uncertainty on our results. Moreover, the sensitivity analyses that we used for our simulations indicate fairly high robustness (i.e., classification didn't change when the simulation parameters were varied). The results, while subject to the selected parameters, are therefore quite probably in favor of DESs. The results also indicate that the benefits of using DES-2s with respect to BMSs decreases when patients at increasingly lower risk of restenosis are included, which is a major indicator in attempting to maximize the anticipated benefits of using DES-2s.

Another limit of our cost-benefit analysis is that we did not consider the cost of re-hospitalization for stent thrombosis and myocardial infarction. Considering that the rates of stent thrombosis and myocardial infarction are lower with DES-2s than BMSs, this would have reinforced our conclusion that DES-2s are cost-effective and should generate higher savings. However, these additional savings should be limited given that stent thrombosis and myocardial infarction are much less frequent than TVR [14], thus leading to a smallest difference in percentage point between DES-2s and BMSs.

## Conclusion

This systematic review of systematic reviews with meta-analyses helps confirm the efficacy of DES-2s compared with BMSs (i.e., better target-vessel revascularization rates). Moreover, no statistically significant difference related to mortality was observed. With respect to myocardial infarction, the DES-2s yielded lower rates at 1 year or less than the BMSs. At more than one year, the Co-Cr-EESs and PC-ZESs appeared to be the most efficacious. In terms of thrombosis rate, the DES-2s seem to perform better than the BMSs, but the difference was not always statistically significant. The Co-Cr-EESs achieved the best results.

Lastly, the unfavorable price difference of DES-2s compared to BMSs (i.e., $190) is largely compensated for by their enhanced efficacy. In our cost–benefit simulations, using DES-2s systematically led to per-patient savings. In our institution, that makes it possible to generalize use to all eligible patients.

## Supporting information

S1 Table PRISMA 2009 checklist. (DOC)
